# Cure of Sickness Attending Pregnancy by Bromide of Potassium

**Published:** 1869-02

**Authors:** 


Dr. D. W. Hodgkins, of Waldoboro, Maine, (*Boston Med. and Surg., Journal*, April 9, 1868,) reports the following case.

Mrs. A. M., fifth pregnancy. Everything went on well until about the sixth month, when she began to have attacks of nausea and vomiting. These attacks increased rapidly in frequency until the nausea become constant. There was a loathing of all food, and if any was taken, it was soon rejected. This condition, after a short time, was accompanied with severe cramps in the limbs and bowels. All the ordinary means for relief were tried successively, but without avail. She became so reduced as to be unable to sit up but a small portion of the time, or to walk across her room without assistance. In this condition, the induction of premature labor seemed the only means to save her life. But before resorting to this I concluded to try the bromide of potassium, and accordingly ordered the following:

| | | |
|---|---|---|
| *R* | Bromidi potass., | oz.ss. |
| | Aquæ font., | f.oz. 4. M |

S. Dessert spoonful once in two hours.

At my next visit, I found all nausea gone. (She had taken the medicine three times in twelve hours.) It did not return again to the same extent, being easily checked by a dose of the medicine. From the taking of the first dose until the end of her term, she never vomited again, but was able to take even hearty food, and gained rapidly in strength. The cramps, which had caused so much suffering, ceased entirely, and she went to the full term without an unfavorable symptom. She was delivered of a fine, healthy boy, and both mother and child did well.

